# DeepDR: a deep learning library for drug response prediction

**DOI:** 10.1093/bioinformatics/btae688

**Published:** 2024-11-18

**Authors:** Zhengxiang Jiang, Pengyong Li

**Affiliations:** School of Computer Science and Technology, Xidian University, Xi’an, Shaanxi 710126, China; School of Electronic Engineering, Xidian University, Xi’an, Shaanxi 710126, China; School of Computer Science and Technology, Xidian University, Xi’an, Shaanxi 710126, China

## Abstract

**Summary:**

Accurate drug response prediction is critical to advancing precision medicine and drug discovery. Recent advances in deep learning (DL) have shown promise in predicting drug response; however, the lack of convenient tools to support such modeling limits their widespread application. To address this, we introduce DeepDR, the first DL library specifically developed for drug response prediction. DeepDR simplifies the process by automating drug and cell featurization, model construction, training, and inference, all achievable with brief programming. The library incorporates three types of drug features along with nine drug encoders, four types of cell features along with nine cell encoders, and two fusion modules, enabling the implementation of up to 135 DL models for drug response prediction. We also explored benchmarking performance with DeepDR, and the optimal models are available on a user-friendly visual interface.

**Availability and implementation:**

DeepDR can be installed from PyPI (https://pypi.org/project/deepdr). The source code and experimental data are available on GitHub (https://github.com/user15632/DeepDR).

## 1 Introduction

Precision medicine aims to deliver tailored therapies for individual tumors at the molecular level. Predicting drug response (DR) (Baptista *et al.* 2021) remains a complex challenge within this field, reflecting the intricate relationship between cancer multi-omics information and treatment efficacy. Accurate DR prediction could significantly contribute to the design of personalized treatments and the improvement of therapeutic outcomes. Deep learning (DL) (LeCun *et al.* 2015), a machine learning approach, has demonstrated considerable promise in identifying complex patterns within biological information, including cancer multi-omics and drug molecules. This potential has spurred its growing application in DR modeling, where it is considered a valuable tool for enhancing understanding and predictive capabilities (Li *et al.* 2021a). However, despite the development of numerous models in this domain, there is still a lack of a unified and generalized framework for model construction and training.

Current DL approaches to DR prediction typically use a structured methodology, consisting of key components such as drug modeling, cell modeling, and fusion modules for prediction generation. Drug modeling aims to effectively represent the chemical properties and potential biological effects of drugs. This is usually achieved by representing the molecular structure in formats conducive to computational processing, such as molecular fingerprints (Li *et al.* 2021a), SMILES (Simplified Molecular Input Line Entry System) (Liu *et al.* 2019), and molecular graphs (Liu *et al.* 2020), followed by learning structural information through models like Deep Neural Networks (DNNs) (Chawla *et al.* 2022), Convolutional Neural Networks (CNNs) (Manica *et al.* 2019), and Graph Neural Networks (GNNs) (Zhang *et al.* 2019). Cell modeling involves processing biological data from cells, including transcriptomics (Chawla *et al.* 2022), genomics (Liu *et al.* 2019), and proteomics (Matlock *et al.* 2018). DL techniques, particularly DNNs (Chawla *et al.* 2022), and CNNs (Manica *et al.* 2019), are leveraged to learn intricate patterns within these features. The fusion module integrates the insights from drug and cell modeling, using DNNs (Chawla *et al.* 2022) or attention mechanisms (Sakellaropoulos *et al.* 2019), to predict drug responses.

DR prediction models have a broad spectrum of applications beyond their primary function. These models can be utilized to predict the pharmacological properties or biological activity of molecules for virtual screening and to analyze omics data for cell classification. The versatility of DL models renders them highly applicable in a range of contexts. For example, clinical researchers investigating the impact of genetic variations on drug responses might use these methodologies to analyze genomic data from patients with specific diseases. Similarly, computational biologists aiming to develop advanced predictive models can leverage diverse datasets to explore various modeling architectures, thereby improving the accuracy of DR predictions. However, implementing these models requires substantial expertise in DL and significant coding efforts. The time-intensive and complexity of adapting to the unique programming interfaces of various open-source tools present nonnegligible challenge requiring resolution.

To address the challenges above, we introduce DeepDR (Deep Drug Response), a Python-based DL library designed for DR prediction. DeepDR incorporates three types of drug features along with nine drug encoders, four types of cell features along with nine cell encoders, as well as two fusion modules. This comprehensive framework supports the implementation of 135 models, catering to clinical researchers and computational biologists with limited programming backgrounds. In addition, we demonstrate the utilization of DeepDR by implementing and validating multiple models on the integrated datasets, which helps to identify the most effective modeling. To further support researchers, we develop a visual interface that enables users without programming expertise to utilize the optimal models.

## 2 DeepDR library

### 2.1 Dataset framework

#### 2.1.1 Featurization

**Figure 1.**
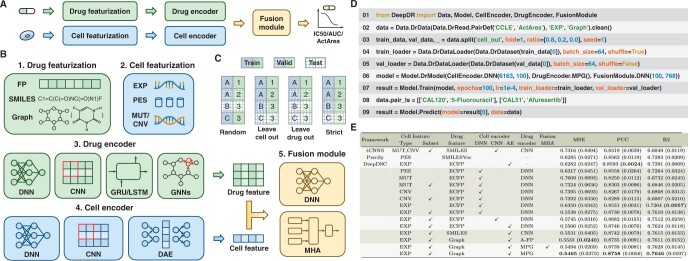
Overview of DeepDR library. (A) The drug and cell are processed through featurization and encoder, and then the drug response is decoded using the fusion module. (B) DeepDR provides drug and cell featurization, encoder, and fusion module. (C) DeepDR provides splitting methods, including random split, leave-cell-out split, leave-drug-out split, and strict split. (D) Programming framework of DeepDR for dataset loading, model implementation, training, and inference. (E) Leave-cell-out performance on the CCLE dataset. Using subset means using features screened on the gene subset, rather than genome-wide features. The values in parentheses are standard deviations.

*Drug featurization.* DeepDR offers three modalities of drug features: FP (Molecular Fingerprints) (Li *et al.* 2021a), SMILES (Simplified Molecular Input Line Entry System) (Liu *et al.* 2019), and molecular graphs (Liu *et al.* 2020) (see Fig. 1B). FP are the binary vector representations of molecules (Rogers and Hahn 2010). SMILES provides a specification for encoding molecules as strings (Weininger 1988). Graphs represent molecules by abstracting atoms as nodes and chemical bonds as edges (Kearnes *et al.* 2016). Details are available in Supplementary Text S1.

*Cell featurization.* DeepDR integrates four modalities of cell features: expression profile (EXP) (Manica *et al.* 2019), pathway enrichment score (PES) (Chawla *et al.* 2022), mutation status (MUT) (Liu *et al.* 2019), and copy number variation (CNV) (Liu *et al.* 2019) (see Fig. 1B). EXP reflects the quantitative expression levels of genes (Heller 2002). PES illuminates the combinatorial implications among genes within specific pathways (Hänzelmann *et al.* 2013). MUT refers to the genetic alterations or variations within specific genes (Stenson *et al.* 2017). CNV represents genomic deletions and duplications observable at the submicroscopic scale (Freeman *et al.* 2006). Given the complexity of processing high-dimensional data, DeepDR provides features screened on gene subsets in addition to genome-wide features (Jia *et al.* 2021). Details are provided in Supplementary Text S2.

#### 2.1.2 Dataset and splitting

DeepDR integrates the Cancer Cell Line Encyclopedia (CCLE) (Barretina *et al.* 2019) and Genomics of Drug Sensitivity in Cancer (GDSC) (Yang *et al.* 2016), and allows users to use their own datasets (see Supplementary Texts S3 and S4). The measurement of drug response is quantified using several parameters: the natural logarithm-transformed IC50 (Half Maximal Inhibitory Concentration), AUC (Area Under the Dose-response Curve), and ActArea (Activity Area). To support the validation, DeepDR incorporates four dataset splitting strategies: common random, leave-cell-out, leave-drug-out, and strict split (Manica *et al.* 2019) (see Fig. 1C). The leave-cell-out split is designed to eliminate any overlap of cells between the training, validation, and test sets. This approach aims to replicate the scenario where the drug response of new cells to existing drugs is evaluated. Similarly, the leave-drug-out split seeks to emulate the response of known cells to novel drugs, while the strict split is designed to simulate the response of novel cells to novel drugs.

### 2.2 Model for DR prediction

Deep learning DR prediction model can be formulated as encoding for drugs and cells and fusion of drug and cell information. In line with this framework, DeepDR has developed three integral modules: the drug encoder, cell encoder, and fusion module. These components are designed to provide the foundation for the flexible construction of predictive models of drug response. The features of drugs and cells are introduced into the encoder. Subsequently, the encoded information is integrated within the fusion module to generate the predicted drug response (see Fig. 1A).

#### 2.2.1 Drug encoder

DeepDR integrates nine encoders tailored to process drug molecular data (see Fig. 1B). These encoders include the DNN (Deep Neural Network) leveraging molecular fingerprints, and architectures such as CNN (Convolutional Neural Network) (Liu *et al.* 2019), GRU (Gated Recurrent Unit) (Dey and Salem 2017), and LSTM (Long Short-Term Memory) (Graves and Graves 2012) that are based on SMILES representations. In addition, it features GCN (Graph Convolutional Network) (Zhang *et al.* 2019), GAT (Graph Attention Network) (Velickovic *et al.* 2017), MPG (Li *et al.* 2021c), AttentiveFP (Xiong *et al.* 2020), and TrimNet (Li *et al.* 2021b) for analyzing molecular graphs. The DNN module encodes the drug as a singular vector, while the other architectures produce a sequence of vectors, with each vector corresponding to a SMILES character or an atom within the molecular graph. The encoders based on SMILES and molecular graphs are integrated with an embedding layer, which is instrumental in generating dense vectors.

#### 2.2.2 Cell encoder

For cell modeling, DeepDR integrates nine encoders: DNN based on EXP, PES, MUT, or CNV (Li *et al.* 2021a); CNN based on EXP, PES, MUT, or CNV (Manica *et al.* 2019); and DAE (Denoising Autoencoder) based on EXP (Chen *et al.* 2022) (see Fig. 1B). The DNN and CNN modules are designed to compress the features of cells into low-dimensional vectors, thus facilitating a more compact and efficient representation of the data. The DAE, on the other hand, is specifically pre-trained to focus on minimizing the reconstruction loss of cell features, utilizing the hidden vectors as the encoding vectors for the cells.

#### 2.2.3 Fusion module

In terms of integrating drug and cell information, DeepDR provides two methods: a DNN based and an MHA (Multi-head Attention)-based framework (see Fig. 1B) (Vaswani *et al.* 2017, Manica *et al.* 2019). The cell encoder is designed to encode the cell as a single vector, while the drug encoder encodes the drug as a single vector or series of vectors. Within the DNN-based framework, a series of vectors can be condensed into a single vector through techniques such as global averaging or maximum pooling. In contrast, the MHA-based approach calculates as follows:

(1) $$\mathrm{Attention}(Q,K,V)=\mathrm{softmax}\left(\frac{QK^{T}}{\sqrt{d_{k}}}\right)V$$

where the cell vector is acting as *Q*. The $d_{k}$ is the dimension of vectors representing the drug, which are considered as the matrices *K* and *V*. This leverages the attention mechanism to effectively extract the information on cell drug interactions into one vector. Both architectures share a common process where the vectors for the drug and cell are either added or concatenated, followed by their introduction into a succession of linear layers for the prediction of drug responses.

## 3 Programming framework of DeepDR

DeepDR streamlines the DR prediction workflow into seven modular components, each thoughtfully structured as a class or function to enhance convenience (see Fig. 1D): (i) Use Data.DrData to construct drug response data, including cell-drug pairs, corresponding drug responses, cell and drug features. (ii) Use .clean() and .split() to clean and split drug response data. (iii) Instantiate the dataset using Data.DrDataset. (iv) Use Data.DrDataLoader to load the dataset for model training or validation. (v) Then Model.DrModel is utilized to construct the DR prediction model. (vi) The model is trained using Model.Train, which concurrently evaluates performance to ensure efficacy. (vii) Finally, Model.Predict is deployed to forecast drug responses, leveraging the knowledge gained from the trained model. DeepDR offers three key metrics: Mean Squared Error (MSE), R-squared (R^2^), and Pearson Correlation Coefficient (PCC).

## 4 Establishing benchmarks via DeepDR

To benchmark drug response prediction, we implemented and evaluated 16 models, including tCNNS (Liu *et al.* 2019), Precily (Chawla *et al.* 2022), and DeepDSC (Li *et al.* 2021a), along with other 13 novel models, on CCLE and GDSC2 datasets. We used leave-cell-out and leave-drug-out splitting strategies to split the datasets into training, validation, and test sets (8:1:1) using three random seeds. Each model was trained for 100 epochs using the MSE loss function, with the learning rate tuned from {0.001, 0.0001, 0.00001}. We report the mean and standard deviation of model performance across the three seeds. Our findings (Fig. 1E and Supplementary Tables S1–S3) highlight three key observations: (i) optimal representations are graphs for drugs and expression profiles for cells. (ii) Predicting the response of novel drugs is a more significant challenge. (iii) Pre-training techniques facilitate accurate prediction of drug response. Further analysis and implementation details can be found in Supplementary Texts S5 and S6 and Supplementary Tables S4–S7. The optimal models developed with DeepDR are available on a visual interface at https://huggingface.co/spaces/user15632/DeepDR.

## Supplementary Material

btae688_Supplementary_Data

## Data Availability

The source code and experimental data are available on GitHub: https://github.com/user15632/DeepDR. Installation of DeepDR involves simply typing “pip install deepdr.”

## References

[btae688-B1] Baptista D , Ferreira PG, Rocha M. Deep learning for drug response prediction in cancer. Brief Bioinform 2021;22:360–79. 31950132 10.1093/bib/bbz171

[btae688-B2] Barretina J , Caponigro G, Stransky N et al Addendum: the cancer cell line encyclopedia enables predictive modelling of anticancer drug sensitivity. Nature 2019;565:E5–6. 30559381 10.1038/s41586-018-0722-x

[btae688-B3] Chawla S , Rockstroh A, Lehman M et al Gene expression based inference of cancer drug sensitivity. Nat Commun 2022;13:5680. 36167836 10.1038/s41467-022-33291-z PMC9515171

[btae688-B4] Chen J , Wang X, Ma A et al Deep transfer learning of cancer drug responses by integrating bulk and single-cell RNA-seq data. Nat Commun 2022;13:6494. 36310235 10.1038/s41467-022-34277-7 PMC9618578

[btae688-B5] Dey R , Salem FM. Gate-variants of gated recurrent unit (GRU) neural networks. In: *2017 IEEE 60th International Midwest Symposium on Circuits and Systems (MWSCAS)*. IEEE, 2017, 1597–600.

[btae688-B6] Freeman JL , Perry GH, Feuk L et al Copy number variation: new insights in genome diversity. Genome Res 2006;16:949–61. 16809666 10.1101/gr.3677206

[btae688-B7] Graves A , Graves A. *Long Short-Term Memory*. *Supervised Sequence Labelling with Recurrent Neural Networks*. New York, USA: Springer, 2012, 37–45.

[btae688-B8] Hänzelmann S , Castelo R, Guinney J. GSVA: gene set variation analysis for microarray and RNA-seq data. BMC Bioinformatics 2013;14:7–15. 23323831 10.1186/1471-2105-14-7 PMC3618321

[btae688-B9] Heller MJ. DNA microarray technology: devices, systems, and applications. Annu Rev Biomed Eng 2002;4:129–53. 12117754 10.1146/annurev.bioeng.4.020702.153438

[btae688-B10] Jia P , Hu R, Pei G et al Deep generative neural network for accurate drug response imputation. Nat Commun 2021;12:1740. 33741950 10.1038/s41467-021-21997-5 PMC7979803

[btae688-B11] Kearnes S , McCloskey K, Berndl M et al Molecular graph convolutions: moving beyond fingerprints. J Comput Aided Mol Des 2016;30:595–608. 27558503 10.1007/s10822-016-9938-8 PMC5028207

[btae688-B12] LeCun Y , Bengio Y, Hinton G. Deep learning. Nature 2015;521:436–44. 26017442 10.1038/nature14539

[btae688-B13] Li M , Wang Y, Zheng R et al Deepdsc: a deep learning method to predict drug sensitivity of cancer cell lines. IEEE/ACM Trans Comput Biol Bioinform2021a;18:575–82. 31150344 10.1109/TCBB.2019.2919581

[btae688-B14] Li P , Li Y, Hsieh C-Y et al Trimnet: learning molecular representation from triplet messages for biomedicine. Brief Bioinform2021b;22:bbaa266. 33147620 10.1093/bib/bbaa266

[btae688-B15] Li P , Wang J, Qiao Y et al An effective self-supervised framework for learning expressive molecular global representations to drug discovery. Brief Bioinform2021c;22:bbab109. 33940598 10.1093/bib/bbab109

[btae688-B16] Liu P , Li H, Li S et al Improving prediction of phenotypic drug response on cancer cell lines using deep convolutional network. BMC Bioinformatics 2019;20:408. 31357929 10.1186/s12859-019-2910-6 PMC6664725

[btae688-B17] Liu Q , Hu Z, Jiang R et al Deepcdr: a hybrid graph convolutional network for predicting cancer drug response. Bioinformatics 2020;36:i911–8. 33381841 10.1093/bioinformatics/btaa822

[btae688-B18] Manica M , Oskooei A, Born J et al Toward explainable anticancer compound sensitivity prediction via multimodal attention-based convolutional encoders. Mol Pharm 2019;16:4797–806. 31618586 10.1021/acs.molpharmaceut.9b00520

[btae688-B19] Matlock K , De Niz C, Rahman R et al Investigation of model stacking for drug sensitivity prediction. BMC Bioinformatics 2018;19:71–33. 29589559 10.1186/s12859-018-2060-2 PMC5872495

[btae688-B20] Rogers D , Hahn M. Extended-connectivity fingerprints. J Chem Inf Model 2010;50:742–54. 20426451 10.1021/ci100050t

[btae688-B21] Sakellaropoulos T , Vougas K, Narang S et al A deep learning framework for predicting response to therapy in cancer. Cell Rep 2019;29:3367–73.e4. 31825821 10.1016/j.celrep.2019.11.017

[btae688-B22] Stenson PD , Mort M, Ball EV et al The human gene mutation database: towards a comprehensive repository of inherited mutation data for medical research, genetic diagnosis and next-generation sequencing studies. Hum Genet 2017;136:665–77. 28349240 10.1007/s00439-017-1779-6 PMC5429360

[btae688-B23] Vaswani A , Shazeer N, Parmar N et al Attention is all you need. Adv Neural Inf Process Syst 2017;30:1–11.

[btae688-B24] Velickovic P , Cucurull G, Casanova A et al Graph attention networks. STAT 2017;1050:10–48550.

[btae688-B25] Weininger D. Smiles, a chemical language and information system. 1. Introduction to methodology and encoding rules. J Chem Inf Comput Sci 1988;28:31–6.

[btae688-B26] Xiong Z , Wang D, Liu X et al Pushing the boundaries of molecular representation for drug discovery with the graph attention mechanism. J Med Chem 2020;63:8749–60. 31408336 10.1021/acs.jmedchem.9b00959

[btae688-B27] Yang W , Lightfoot H, Bignell G et al Genomics of drug sensitivity in cancer (GDSC): a resource for biomarker discovery in cancer cells. Eur J Cancer 2016;69:S82. 10.1016/S0959-8049(16)32839-8 PMC3531057 23180760

[btae688-B28] Zhang S , Tong H, Xu J et al Graph convolutional networks: a comprehensive review. Comput Soc Netw 2019;6:11–23. 37915858 10.1186/s40649-019-0069-y PMC10615927

