# Supplementary material for: DeepDR: a deep learning library for drug response prediction
Source: Bioinformatics. 2024 Nov 18;40(12):btae688. doi: 10.1093/bioinformatics/btae688 (PMC11629690; doi:10.1093/bioinformatics/btae688)
Supplement: btae688_Supplementary_Data [file btae688_supplementary_data.pdf]

DeepDR: a deep learning library for drug response prediction

Zhengxiang Jiang[1,2] and Pengyong Li[1,*]

1 School of Computer Science and Technology, Xidian University, 710126, Xi'an, Shaanxi, China

2 School of Electronic Engineering, Xidian University, 710126, Xi'an, Shaanxi, China

* Corresponding author. lipengyong@xidian.edu.cn

1 Supplementary Text

Text S1: Details of drug featurization

In DeepDR, the molecular fingerprints and SMILES (Simplified Molecular Input Line Entry System) of drugs were obtained through the rdkit library, and the molecular graphs were obtained through the mol_to_graph_data_obj_complex function adopted in MolGNet[1]. For the molecular graph, the atom is represented by atomic number, formal charge, chirality, hybridization, number of hydrogen atoms, number of implicit valence bonds, degree, and aromaticity, and the bond is represented by bond type, bond direction, conjugacy, whether the it is in the ring, stereochemistry.

Text S2: Details of cell featurization

DeepDR maintains dictionaries that map cell names to cell features. The original data of cell features were obtained from the Genomics of Drug Sensitivity in Cancer (GDSC) database (https://www.cancerrxgene.org/) and preprocessed. Expression profiles (EXP) obtained by microarray technique were RMA (Robust Multi-array Average) normalized and standardized[2]. For pathway enrichment scores (PES), we calculated the GSVA (Gene Set Variation Analysis) scores using the GSVA R package based on the expression profiles and the c2 collection of canonical pathways (MSigDB.CP.v.6.1) consisting of 1,329 gene sets from MSigDB (Molecular Signatures Database) [3], and then the PES were standardized. For mutation status (MUT), only non-silent mutations were retained, and wild types were coded as 0 and mutation types as 1. For copy number variations (CNV), copy-neutrals were coded as 0 and deletions or amplifications were coded as 1[2]. For features screened on gene subsets, we used a subset of 6,163 genes that were most variably expressed across cell lines[4]. For cases where data for the corresponding gene is missing, 0 padding is used for MUT and CNV, and the mean (approximately 2) padding is used for EXP. In particular, the screened and padded EXP is then standardized. The standardized techniques used is z-score, that is, minus the mean value and then divide by the standard deviation.

Text S3: Details of integrated dataset

The CCLE dataset contains 11,670 cell-drug pairs (including 504 cells and 24 drugs), GDSC1 contains 333,161 (including 970 cells and 378 drugs), and GDSC2 contains 242,036 (including 969 cells and 286 drugs).

Text S4: Guidance on using your own dataset

The steps required for users to train models with their datasets are as follows: (i) Prepare raw data. First, prepare the gene subset. Users need to prepare a gbk encoding txt file. Each row in the file should be a gene name. Secondly, Prepare the cell data. Users need to prepare a csv file with column separators of ",". The first column in the file should be the gene name, the first row should be the

cell name. Finally, prepare the response data. Users need to prepare a csv file with column separators of ",". The first row is the header, the second row to the last row are cell-drug pairs, where the first column is the cell name, the second column is the drug name, and the third column is the drug response. (ii) Proceed feature extraction. Use Data.DrRead.FeatCell to prepare the cell feature. Use Data.DrRead.FeatDrug to prepare the drug feature. Use Data.DrRead.PairCSV to read the response data. (iii) Use Data.DrData to construct data. More detailed guidance can be found in the documentation (https://deepdr.readthedocs.io/en/latest/).

Text S5: Results of the benchmark test

Optimal representations: graphs for drugs and expression profiles for cells. In deep learning-based drug response prediction, powerful representations of drugs and cells are critical for effective modeling. After extensive experiments, it has become clear that representing drugs as graphs, where nodes represented atoms and edges represented bonds, most effectively characterized complex molecular structures and chemical properties of drugs. On the other hand, the optimal representations of cells were gene expression profiles, which encapsulated the transcriptional levels of thousands of genes within the cellular environment, reflecting the cell's physiological state and potential vulnerability to drug intervention. Combining these representations allowed deep learning models to effectively learn the complex interactions between drugs and cells, leading to more accurate drug response prediction.

Predicting the response of novel drugs is a more significant challenge. Precision medicine and drug discovery require models that enable accurate predictions of responses to novel drugs or novel cells. Catering to this demand, we validated the model's predictive performance under two strategies: leave-cell-out and leave-drug-out. Results (see Figure 1E and Table S1-S3) indicated that predictive accuracy and robustness were significantly lower when using leave-drug-out strategies than leave-cell-out strategies. This finding suggested that predicting responses to novel drugs was more challenging than predicting responses of novel cells. One possible explanation for this discrepancy was that the number of drugs in the dataset was relatively small compared to the number of cells, which may limit the ability of the model to generalize to unlearned compounds effectively.

Pre-training techniques facilitate accurate prediction of drug response. Pre-training techniques are widely employed in machine learning to train models on large, generic datasets before fine-tuning their parameters for specific tasks. In DeepDR, we introduced two pre-trained models: the drug encoder MPG and the cell encoder DAE. As demonstrated in Figure 1E and Table S1-S3, the pre-trained drug encoder exhibited a significant performance advantage over other models. When evaluated on the CCLE dataset, the pre-trained cell encoder outperformed others, whereas untrained cell encoders showed better results on the GDSC2 dataset. This suggested that pre-training may reduce the risk of overfitting in smaller datasets like CCLE. In comparison, direct training on larger datasets like GDSC2 may allow models to learn complex relationships without pre-training. Notably, the benefits of pre-training were more pronounced for drug encoders than for cell encoders, potentially due to the availability of 11 million drug molecule samples for pre-training, compared to only 975 samples for cells, which limited the ability to learn comprehensive representations.

Text S6: Details of the benchmark test

In the benchmark test, due to the absence of some cell or drug features, 7,523 cell-drug pairs were used in the CCLE dataset, including 327 cells and 24 drugs, and 118,955 cell-drug pairs were used in the GDSC2 dataset, including 570 cells and 228 drugs. The DeepDR version used is 0.1, which can be downloaded from https://drive.google.com/file/d/1usL_HFmCfndN4hkHq97CR4Lj1JaxiMm_/view?usp=sharing. The scripts used and the resulting files can be obtained at https://github.com/user15632/DeepDR/tree/main/results. The performance of models at different learning rates in the benchmark test is provided in Table S4-S7.

2 Supplementary Table

Table S1: Leave-drug-out performance on the CCLE dataset

Framework	Cell feature		Drug	Cell encoder			Drug	Fusion	MSE	PCC	R2
	Type	[1]Subset	feature	DNN	CNN	AE	encoder	MHA			
tCNNS	MUT,CNV	✓	SMILES		✓		CNN		1.2489 (0.4240)	0.2472 (0.0677)	-0.4920 (0.5285)
Precily	PES		SMILESVec				–		1.2540 (0.8432)	0.1769 (0.1582)	-0.2057 (0.0595)
DeepDSC	EXP		ECFP			✓	–		0.8405 (0.2366)	0.4095 (0.1616)	-0.0147 (0.3044)
	PES		ECFP	✓			DNN		0.8718 (0.4191)	0.3772 (0.0896)	0.0560 (0.1413)
	MUT		ECFP	✓			DNN		0.8888 (0.5185)	0.3688 **(0.0428)**	0.0918 (0.0787)
	MUT	✓	ECFP	✓			DNN		0.8504 (0.5230)	0.4272 (0.0574)	0.1525 (0.0326)
	CNV		ECFP	✓			DNN		0.8738 (0.5330)	0.3953 (0.0742)	0.1267 (0.0412)
	CNV	✓	ECFP	✓			DNN		0.8273 (0.3179)	0.3885 (0.1453)	0.0545 (0.2129)
	EXP	✓	ECFP	✓			DNN		0.8450 (0.5180)	0.4361 (0.0627)	0.1562 **(0.0260)**
	EXP		ECFP	✓			DNN		0.7338 (0.3212)	0.4785 (0.1391)	0.1869 (0.1450)
	EXP		ECFP		✓		DNN		0.9997 (0.4551)	0.3464 (0.0494)	-0.1088 (0.2569)
	EXP		ECFP			✓	DNN		0.6917 (0.3278)	0.5195 (0.1557)	**0.2515** (0.1185)
	EXP		SMILES			✓	CNN		1.0847 (0.6312)	0.1961 (0.1435)	-0.1020 (0.0831)
	EXP		Graph			✓	A-FP		0.8190 (0.4526)	**0.5963** (0.1635)	0.1491 (0.0790)
	EXP		Graph			✓	MPG		0.6495 **(0.0725)**	0.3483 (0.3705)	0.1282 (0.3689)
	EXP		Graph			✓	MPG	✓	**0.6311** (0.1106)	0.4645 (0.2314)	0.1989 (0.2838)

[1]Using subset means using features screened on the gene subset, rather than genome-wide features.

Table S2: Leave-cell-out performance on the GDSC2 dataset

Framework	Cell feature		Drug	Cell encoder			Drug	Fusion	MSE	PCC	R2
	Type	[1]Subset	feature	DNN	CNN	AE	encoder	MHA			
tCNNS	MUT,CNV	✓	SMILES		✓		CNN		2.2774 (0.1589)	0.8473 (0.0104)	0.7123 (0.0210)
Precily	PES		SMILESVec				–		2.1597 (0.1249)	0.8670 (0.0082)	0.7272 (0.0168)
DeepDSC	EXP		ECFP			✓	–		2.0226 (0.1130)	0.8647 (0.0073)	0.7445 (0.0152)
	PES		ECFP	✓			DNN		1.8244 **(0.0892)**	0.8792 **(0.0057)**	0.7695 **(0.0122)**
	MUT		ECFP	✓			DNN		2.3763 (0.1698)	0.8402 (0.0087)	0.6998 (0.0229)
	MUT	✓	ECFP	✓			DNN		2.5406 (0.1251)	0.8302 (0.0074)	0.6791 (0.0173)
	CNV		ECFP	✓			DNN		2.2745 (0.2227)	0.8455 (0.0163)	0.7126 (0.0292)
	CNV	✓	ECFP	✓			DNN		2.3392 (0.2518)	0.8432 (0.0168)	0.7045 (0.0329)
	EXP		ECFP	✓			DNN		2.0050 (0.1461)	0.8689 (0.0076)	0.7467 (0.0196)
	EXP	✓	ECFP	✓			DNN		1.8076 (0.1838)	0.8819 (0.0142)	0.7716 (0.0242)
	EXP	✓	ECFP			✓	DNN		1.8696 (0.1569)	0.8779 (0.0107)	0.7638 (0.0209)
	EXP	✓	ECFP		✓		DNN		1.7145 (0.2294)	0.8860 (0.0164)	0.7833 (0.0300)
	EXP	✓	SMILES		✓		CNN		1.6475 (0.1859)	0.8915 (0.0122)	0.7918 (0.0244)
	EXP	✓	Graph		✓		A-FP		2.7031 (0.3012)	0.8354 (0.0214)	0.6587 (0.0375)
	EXP	✓	Graph		✓		MPG		1.6282 (0.1634)	0.8919 (0.0113)	0.7943 (0.0216)
	EXP	✓	Graph		✓		MPG	✓	**1.6238** (0.1716)	**0.8921** (0.0120)	**0.7948** (0.0226)

[1]Using subset means using features screened on the gene subset, rather than genome-wide features.

Table S3: Leave-drug-out performance on the GDSC2 dataset

Framework	Cell feature		Drug	Cell encoder			Drug	Fusion	MSE	PCC	R2
	Type	[1]Subset	feature	DNN	CNN	AE	encoder	MHA			
tCNNS	MUT,CNV	✓	SMILES		✓		CNN		5.4641 (1.8772)	0.4923 (0.0736)	0.2116 (0.1074)
Precily	PES		SMILESVec				–		5.7913 (1.0993)	0.5044 (0.1157)	0.1374 (0.0865)
DeepDSC	EXP		ECFP			✓	–		5.6345 (0.9334)	0.4741 **(0.0343)**	0.1591 **(0.0285)**
	PES		ECFP	✓			DNN		5.1704 (0.4672)	0.4910 (0.1544)	0.2029 (0.1781)
	MUT		ECFP	✓			DNN		5.2378 (0.7328)	0.4753 (0.2060)	0.1816 (0.2441)
	MUT	✓	ECFP	✓			DNN		5.9389 (1.0590)	0.4251 (0.0704)	0.1151 (0.0309)
	CNV		ECFP	✓			DNN		5.0854 (0.5315)	0.4842 (0.1880)	0.2126 (0.1958)
	CNV	✓	ECFP	✓			DNN		5.3154 (0.5148)	0.4858 (0.1385)	0.1928 (0.1121)
	EXP	✓	ECFP	✓			DNN		5.8825 (0.6335)	0.4629 (0.1676)	0.1080 (0.1216)
	EXP		ECFP	✓			DNN		4.8822 (0.2762)	0.5136 (0.1512)	0.2513 (0.1350)
	EXP		ECFP		✓		DNN		5.1636 **(0.2154)**	0.5014 (0.1278)	0.2073 (0.1425)
	EXP		ECFP			✓	DNN		5.3094 (0.4527)	0.4363 (0.1630)	0.1914 (0.1222)
	EXP		SMILES	✓			CNN		5.1819 (0.2681)	0.5251 (0.1136)	0.1974 (0.1793)
	EXP		Graph	✓			A-FP		4.6593 (0.3558)	0.5320 (0.1091)	0.2897 (0.1085)
	EXP		Graph	✓			MPG	✓	4.9249 (0.2789)	0.5099 (0.0964)	0.2492 (0.1053)
	EXP		Graph	✓			MPG		**4.3864** (0.4431)	**0.6009** (0.0446)	**0.3373** (0.0620)

[1]Using subset means using features screened on the gene subset, rather than genome-wide features.

Table S4: Leave-cell-out performance on CCLE with different learning rates

Framework	Random seed	Learning rate	MSE
tCNNS	1	1e-03	0.6740
		1e-04	0.7536
		1e-05	0.7754
	10	1e-03	0.6543
		1e-04	0.6780
		1e-05	0.6835
	100	1e-03	0.7129
		1e-04	0.7532
		1e-05	0.7620
Precily	1	1e-03	0.6222
		1e-04	0.6391
		1e-05	0.6591
	10	1e-03	0.4983
		1e-04	0.5549
		1e-05	0.5752
	100	1e-03	0.6416
		1e-04	0.6909
		1e-05	0.6993
DeepDSC	1	1e-03	0.5729
		1e-04	0.6814
		1e-05	0.7078
	10	1e-03	0.5419
		1e-04	0.6188
		1e-05	0.6292
	100	1e-03	0.5268
		1e-04	0.5867
		1e-05	0.6073
Drug feature: ECFP<br>Cell feature: PES<br>Drug encoder: DNN<br>Cell encoder: DNN<br>Fusion module: DNN	1	1e-03	0.6630
		1e-04	0.7764
		1e-05	0.8315
	10	1e-03	0.5240
		1e-04	0.5816
		1e-05	0.5976
	100	1e-03	0.6027
		1e-04	0.6528
		1e-05	0.6669

Continued Table S4: Leave-cell-out performance on CCLE with different learning rates

Framework	Random seed	Learning rate	MSE
Drug feature: ECFP<br>Cell feature: MUT_{all}<br>Drug encoder: DNN<br>Cell encoder: DNN<br>Fusion module: DNN	1	1e-03	0.7407
		1e-04	0.7330
		1e-05	0.7536
	10	1e-03	0.6707
		1e-04	0.8159
		1e-05	0.8498
	100	1e-03	0.7078
		1e-04	0.7486
		1e-05	0.7865
Drug feature: ECFP<br>Cell feature: MUT_{sub}<br>Drug encoder: DNN<br>Cell encoder: DNN<br>Fusion module: DNN	1	1e-03	0.7098
		1e-04	0.7457
		1e-05	0.7662
	10	1e-03	0.6943
		1e-04	0.7250
		1e-05	0.7676
	100	1e-03	0.7019
		1e-04	0.7347
		1e-05	0.7480
Drug feature: ECFP<br>Cell feature: CNV_{all}<br>Drug encoder: DNN<br>Cell encoder: DNN<br>Fusion module: DNN	1	1e-03	0.7440
		1e-04	0.8278
		1e-05	0.8769
	10	1e-03	0.6050
		1e-04	0.6739
		1e-05	0.6865
	100	1e-03	0.7165
		1e-04	0.7667
		1e-05	0.8012
Drug feature: ECFP<br>Cell feature: CNV_{sub}<br>Drug encoder: DNN<br>Cell encoder: DNN<br>Fusion module: DNN	1	1e-03	0.7251
		1e-04	0.8173
		1e-05	0.8724
	10	1e-03	0.6147
		1e-04	0.6697
		1e-05	0.6933
	100	1e-03	0.6916
		1e-04	0.7455
		1e-05	0.7850

Continued Table S4: Leave-cell-out performance on CCLE with different learning rates

Framework	Random seed	Learning rate	MSE
Drug feature: ECFP<br>Cell feature: EXP_{all}<br>Drug encoder: DNN<br>Cell encoder: DNN<br>Fusion module: DNN	1	1e-03	0.6602
		1e-04	0.6904
		1e-05	0.7180
	10	1e-03	0.5251
		1e-04	0.5429
		1e-05	0.5461
	100	1e-03	0.6420
		1e-04	0.6831
		1e-05	0.6941
Drug feature: ECFP<br>Cell feature: EXP_{sub}<br>Drug encoder: DNN<br>Cell encoder: DNN<br>Fusion module: DNN	1	1e-03	0.5566
		1e-04	0.5664
		1e-05	0.5720
	10	1e-03	0.4945
		1e-04	0.5127
		1e-05	0.5311
	100	1e-03	0.6029
		1e-04	0.6037
		1e-05	0.6159
Drug feature: ECFP<br>Cell feature: EXP_{sub}<br>Drug encoder: DNN<br>Cell encoder: CNN<br>Fusion module: DNN	1	1e-03	0.5690
		1e-04	0.6029
		1e-05	0.6227
	10	1e-03	0.4844
		1e-04	0.5247
		1e-05	0.5336
	100	1e-03	0.5735
		1e-04	0.6244
		1e-05	0.6403
Drug feature: ECFP<br>Cell feature: EXP_{sub}<br>Drug encoder: DNN<br>Cell encoder: DAE<br>Fusion module: DNN	1	1e-03	0.5549
		1e-04	0.5742
		1e-05	0.5954
	10	1e-03	0.5048
		1e-04	0.5103
		1e-05	0.5175
	100	1e-03	0.5952
		1e-04	0.6007
		1e-05	0.6102

Continued Table S4: Leave-cell-out performance on CCLE with different learning rates

Framework	Random seed	Learning rate	MSE
Drug feature: SMILES<br>Cell feature: EXP_{sub}<br>Drug encoder: CNN<br>Cell encoder: DAE<br>Fusion module: DNN	1	1e-03	0.5331
		1e-04	0.5419
		1e-05	0.5552
	10	1e-03	0.5030
		1e-04	0.5655
		1e-05	0.5901
	100	1e-03	0.6047
		1e-04	0.6048
		1e-05	0.6191
Drug feature: Graph<br>Cell feature: EXP_{sub}<br>Drug encoder: A-FP<br>Cell encoder: DAE<br>Fusion module: DNN	1	1e-03	0.5341
		1e-04	0.5558
		1e-05	0.5722
	10	1e-03	0.5096
		1e-04	0.5130
		1e-05	0.5173
	100	1e-03	0.5985
		1e-04	0.6318
		1e-05	0.6451
Drug feature: Graph<br>Cell feature: EXP_{sub}<br>Drug encoder: MPG<br>Cell encoder: DAE<br>Fusion module: DNN	1	1e-03	0.5567
		1e-04	0.5743
		1e-05	0.5914
	10	1e-03	0.5044
		1e-04	0.5160
		1e-05	0.5288
	100	1e-03	0.5609
		1e-04	0.5741
		1e-05	0.5911
Drug feature: Graph<br>Cell feature: EXP_{sub}<br>Drug encoder: MPG<br>Cell encoder: DAE<br>Fusion module: MHA	1	1e-03	0.5337
		1e-04	0.5718
		1e-05	0.6009
	10	1e-03	0.4888
		1e-04	0.5292
		1e-05	0.5408
	100	1e-03	0.5763
		1e-04	0.6024
		1e-05	0.6194

Table S5: Leave-drug-out performance on CCLE with different learning rates

Framework	Random seed	Learning rate	MSE
tCNNS	1	1e-03	0.5275
		1e-04	0.7735
		1e-05	0.8195
	10	1e-03	1.0819
		1e-04	1.6444
		1e-05	1.7075
	100	1e-03	2.5363
		1e-04	2.7803
		1e-05	2.8086
Precily	1	1e-03	0.8937
		1e-04	1.0388
		1e-05	1.0738
	10	1e-03	0.3644
		1e-04	0.3832
		1e-05	0.3937
	100	1e-03	1.0119
		1e-04	1.3261
		1e-05	1.4032
DeepDSC	1	1e-03	0.7575
		1e-04	0.8800
		1e-05	0.9261
	10	1e-03	0.5103
		1e-04	0.6858
		1e-05	0.7526
	100	1e-03	1.8082
		1e-04	1.8198
		1e-05	1.8538
Drug feature: ECFP<br>Cell feature: PES<br>Drug encoder: DNN<br>Cell encoder: DNN<br>Fusion module: DNN	1	1e-03	0.4149
		1e-04	0.9976
		1e-05	1.0206
	10	1e-03	0.4389
		1e-04	0.5431
		1e-05	0.5794
	100	1e-03	2.1255
		1e-04	2.5584
		1e-05	2.5949

Continued Table S5: Leave-drug-out performance on CCLE with different learning rates

Framework	Random seed	Learning rate	MSE
Drug feature: ECFP<br>Cell feature: MUT_{all}<br>Drug encoder: DNN<br>Cell encoder: DNN<br>Fusion module: DNN	1	1e-03	0.7948
		1e-04	0.9627
		1e-05	0.9979
	10	1e-03	0.4244
		1e-04	0.4685
		1e-05	0.4822
	100	1e-03	2.2571
		1e-04	2.4013
		1e-05	2.4043
Drug feature: ECFP<br>Cell feature: MUT_{sub}<br>Drug encoder: DNN<br>Cell encoder: DNN<br>Fusion module: DNN	1	1e-03	0.4379
		1e-04	0.8886
		1e-05	0.9154
	10	1e-03	0.4070
		1e-04	0.4635
		1e-05	0.4850
	100	1e-03	2.1769
		1e-04	2.2032
		1e-05	2.2473
Drug feature: ECFP<br>Cell feature: CNV_{all}<br>Drug encoder: DNN<br>Cell encoder: DNN<br>Fusion module: DNN	1	1e-03	0.4756
		1e-04	0.8803
		1e-05	0.9418
	10	1e-03	0.5078
		1e-04	0.6926
		1e-05	0.7696
	100	1e-03	1.9277
		1e-04	2.3325
		1e-05	2.3955
Drug feature: ECFP<br>Cell feature: CNV_{sub}<br>Drug encoder: DNN<br>Cell encoder: DNN<br>Fusion module: DNN	1	1e-03	0.4346
		1e-04	0.9558
		1e-05	0.9736
	10	1e-03	0.4099
		1e-04	0.4554
		1e-05	0.4577
	100	1e-03	2.0093
		1e-04	2.6372
		1e-05	2.6964

Continued Table S5: Leave-drug-out performance on CCLE with different learning rates

Framework	Random seed	Learning rate	MSE
Drug feature: ECFP<br>Cell feature: EXPall<br>Drug encoder: DNN<br>Cell encoder: DNN<br>Fusion module: DNN	1	1e-03	0.7636
		1e-04	0.9461
		1e-05	1.0078
	10	1e-03	0.4498
		1e-04	0.4668
		1e-05	0.4672
	100	1e-03	1.8640
		1e-04	1.9870
		1e-05	1.9919
Drug feature: ECFP<br>Cell feature: EXPsub<br>Drug encoder: DNN<br>Cell encoder: DNN<br>Fusion module: DNN	1	1e-03	0.5140
		1e-04	0.8476
		1e-05	0.8667
	10	1e-03	0.4112
		1e-04	0.4645
		1e-05	0.4927
	100	1e-03	2.2413
		1e-04	2.4228
		1e-05	2.4773
Drug feature: ECFP<br>Cell feature: EXPall<br>Drug encoder: DNN<br>Cell encoder: CNN<br>Fusion module: DNN	1	1e-03	0.6512
		1e-04	0.7472
		1e-05	0.7191
	10	1e-03	0.5116
		1e-04	1.0811
		1e-05	1.2434
	100	1e-03	2.0742
		1e-04	2.2587
		1e-05	2.4313
Drug feature: ECFP<br>Cell feature: EXPall<br>Drug encoder: DNN<br>Cell encoder: DAE<br>Fusion module: DNN	1	1e-03	0.7679
		1e-04	0.8749
		1e-05	0.9442
	10	1e-03	0.4502
		1e-04	0.4701
		1e-05	0.4678
	100	1e-03	2.0635
		1e-04	2.1232
		1e-05	2.1624

Continued Table S5: Leave-drug-out performance on CCLE with different learning rates

Framework	Random seed	Learning rate	MSE
Drug feature: SMILES<br>Cell feature: EXP_{all}<br>Drug encoder: CNN<br>Cell encoder: DAE<br>Fusion module: DNN	1	1e-03	0.8309
		1e-04	0.9628
		1e-05	0.9893
	10	1e-03	0.3969
		1e-04	0.4149
		1e-05	0.4141
	100	1e-03	1.7313
		1e-04	1.9952
		1e-05	2.0225
Drug feature: Graph<br>Cell feature: EXP_{all}<br>Drug encoder: A-FP<br>Cell encoder: DAE<br>Fusion module: DNN	1	1e-03	0.7678
		1e-04	0.8143
		1e-05	0.9435
	10	1e-03	0.4211
		1e-04	0.4899
		1e-05	0.4996
	100	1e-03	0.5979
		1e-04	0.5735
		1e-05	1.3722
Drug feature: Graph<br>Cell feature: EXP_{all}<br>Drug encoder: MPG<br>Cell encoder: DAE<br>Fusion module: DNN	1	1e-03	0.7372
		1e-04	0.7515
		1e-05	0.7705
	10	1e-03	0.4261
		1e-04	0.5080
		1e-05	0.5183
	100	1e-03	1.7670
		1e-04	2.1456
		1e-05	2.2021
Drug feature: Graph<br>Cell feature: EXP_{all}<br>Drug encoder: MPG<br>Cell encoder: DAE<br>Fusion module: MHA	1	1e-03	0.7595
		1e-04	0.8533
		1e-05	0.8661
	10	1e-03	0.3901
		1e-04	0.3896
		1e-05	0.3942
	100	1e-03	0.8471
		1e-04	1.2449
		1e-05	1.3397

Table S6: Leave-cell-out performance on GDSC2 with different learning rates

Framework	Random seed	Learning rate	MSE
tCNNS	1	1e-03	0.6740
		1e-04	0.7536
		1e-05	0.7754
	10	1e-03	0.6543
		1e-04	0.6780
		1e-05	0.6835
	100	1e-03	0.7129
		1e-04	0.7532
		1e-05	0.7620
Precily	1	1e-03	0.6222
		1e-04	0.6391
		1e-05	0.6591
	10	1e-03	0.4983
		1e-04	0.5549
		1e-05	0.5752
	100	1e-03	0.6416
		1e-04	0.6909
		1e-05	0.6993
DeepDSC	1	1e-03	0.5729
		1e-04	0.6814
		1e-05	0.7078
	10	1e-03	0.5419
		1e-04	0.6188
		1e-05	0.6292
	100	1e-03	0.5268
		1e-04	0.5867
		1e-05	0.6073
Drug feature: ECFP<br>Cell feature: PES<br>Drug encoder: DNN<br>Cell encoder: DNN<br>Fusion module: DNN	1	1e-03	0.6630
		1e-04	0.7764
		1e-05	0.8315
	10	1e-03	0.5240
		1e-04	0.5816
		1e-05	0.5976
	100	1e-03	0.6027
		1e-04	0.6528
		1e-05	0.6669

Continued Table S6: Leave-cell-out performance on GDSC2 with different learning rates

Framework	Random seed	Learning rate	MSE
Drug feature: ECFP<br>Cell feature: MUT_{all}<br>Drug encoder: DNN<br>Cell encoder: DNN<br>Fusion module: DNN	1	1e-03	0.7407
		1e-04	0.7330
		1e-05	0.7536
	10	1e-03	0.6707
		1e-04	0.8159
		1e-05	0.8498
	100	1e-03	0.7078
		1e-04	0.7486
		1e-05	0.7865
Drug feature: ECFP<br>Cell feature: MUT_{sub}<br>Drug encoder: DNN<br>Cell encoder: DNN<br>Fusion module: DNN	1	1e-03	0.7098
		1e-04	0.7457
		1e-05	0.7662
	10	1e-03	0.6943
		1e-04	0.7250
		1e-05	0.7676
	100	1e-03	0.7019
		1e-04	0.7347
		1e-05	0.7480
Drug feature: ECFP<br>Cell feature: CNV_{all}<br>Drug encoder: DNN<br>Cell encoder: DNN<br>Fusion module: DNN	1	1e-03	0.7440
		1e-04	0.8278
		1e-05	0.8769
	10	1e-03	0.6050
		1e-04	0.6739
		1e-05	0.6865
	100	1e-03	0.7165
		1e-04	0.7667
		1e-05	0.8012
Drug feature: ECFP<br>Cell feature: CNV_{sub}<br>Drug encoder: DNN<br>Cell encoder: DNN<br>Fusion module: DNN	1	1e-03	0.7251
		1e-04	0.8173
		1e-05	0.8724
	10	1e-03	0.6147
		1e-04	0.6697
		1e-05	0.6933
	100	1e-03	0.6916
		1e-04	0.7455
		1e-05	0.7850

Continued Table S6: Leave-cell-out performance on GDSC2 with different learning rates

Framework	Random seed	Learning rate	MSE
Drug feature: ECFP<br>Cell feature: EXP_{all}<br>Drug encoder: DNN<br>Cell encoder: DNN<br>Fusion module: DNN	1	1e-03	2.4058
		1e-04	2.4678
		1e-05	2.4933
	10	1e-03	2.0916
		1e-04	2.1896
		1e-05	2.2137
	100	1e-03	2.1436
		1e-04	2.2131
		1e-05	2.2366
Drug feature: ECFP<br>Cell feature: EXP_{sub}<br>Drug encoder: DNN<br>Cell encoder: DNN<br>Fusion module: DNN	1	1e-03	2.5122
		1e-04	2.6964
		1e-05	2.8023
	10	1e-03	2.1777
		1e-04	2.2161
		1e-05	2.2856
	100	1e-03	2.0077
		1e-04	2.0968
		1e-05	2.1704
Drug feature: ECFP<br>Cell feature: EXP_{sub}<br>Drug encoder: DNN<br>Cell encoder: CNN<br>Fusion module: DNN	1	1e-03	2.0322
		1e-04	2.1631
		1e-05	2.2102
	10	1e-03	1.9368
		1e-04	2.0399
		1e-05	2.0765
	100	1e-03	1.8442
		1e-04	1.9638
		1e-05	2.0012
Drug feature: ECFP<br>Cell feature: EXP_{sub}<br>Drug encoder: DNN<br>Cell encoder: DAE<br>Fusion module: DNN	1	1e-03	2.1080
		1e-04	2.0925
		1e-05	2.1116
	10	1e-03	1.9827
		1e-04	2.0125
		1e-05	2.0298
	100	1e-03	1.8343
		1e-04	1.8323
		1e-05	1.8523

Continued Table S6: Leave-cell-out performance on GDSC2 with different learning rates

Framework	Random seed	Learning rate	MSE
Drug feature: SMILES<br>Cell feature: EXPsub<br>Drug encoder: CNN<br>Cell encoder: CNN<br>Fusion module: DNN	1	1e-03	2.5286
		1e-04	2.5363
		1e-05	2.5545
	10	1e-03	2.2678
		1e-04	2.2952
		1e-05	2.3249
	100	1e-03	2.3922
		1e-04	2.3783
		1e-05	2.4018
Drug feature: Graph<br>Cell feature: EXPsub<br>Drug encoder: A-FP<br>Cell encoder: CNN<br>Fusion module: DNN	1	1e-03	2.6070
		1e-04	2.7219
		1e-05	2.7350
	10	1e-03	2.3092
		1e-04	2.3900
		1e-05	2.4140
	100	1e-03	2.4838
		1e-04	2.5894
		1e-05	2.5993
Drug feature: Graph<br>Cell feature: EXPsub<br>Drug encoder: MPG<br>Cell encoder: CNN<br>Fusion module: DNN	1	1e-03	2.5804
		1e-04	2.7218
		1e-05	2.7804
	10	1e-03	2.0867
		1e-04	2.1143
		1e-05	2.1436
	100	1e-03	2.3895
		1e-04	2.6141
		1e-05	2.6903
Drug feature: Graph<br>Cell feature: EXPsub<br>Drug encoder: MPG<br>Cell encoder: CNN<br>Fusion module: MHA	1	1e-03	2.5040
		1e-04	2.5794
		1e-05	2.6029
	10	1e-03	2.2111
		1e-04	2.2392
		1e-05	2.2419
	100	1e-03	2.4671
		1e-04	2.5842
		1e-05	2.6147

Table S7: Leave-drug-out performance on GDSC2 with different learning rates

Framework	Random seed	Learning rate	MSE
tCNNS	1	1e-03	4.8288
		1e-04	5.1531
		1e-05	5.2452
	10	1e-03	6.2554
		1e-04	7.1845
		1e-05	7.2352
	100	1e-03	4.9037
		1e-04	5.7384
		1e-05	5.8581
Precily	1	1e-03	5.3867
		1e-04	5.9230
		1e-05	6.0017
	10	1e-03	9.8574
		1e-04	10.8722
		1e-05	11.1433
	100	1e-03	6.9207
		1e-04	8.2891
		1e-05	8.5322
DeepDSC	1	1e-03	5.0592
		1e-04	5.5752
		1e-05	5.6766
	10	1e-03	8.3137
		1e-04	8.3108
		1e-05	8.3244
	100	1e-03	5.3093
		1e-04	5.6051
		1e-05	5.6900
Drug feature: ECFP<br>Cell feature: PES<br>Drug encoder: DNN<br>Cell encoder: DNN<br>Fusion module: DNN	1	1e-03	4.5657
		1e-04	4.6805
		1e-05	4.7048
	10	1e-03	7.6534
		1e-04	8.9443
		1e-05	8.9269
	100	1e-03	5.1969
		1e-04	6.8242
		1e-05	7.1882

Continued Table S7: Leave-drug-out performance on GDSC2 with different learning rates

Framework	Random seed	Learning rate	MSE
Drug feature: ECFP<br>Cell feature: MUT_{all}<br>Drug encoder: DNN<br>Cell encoder: DNN<br>Fusion module: DNN	1	1e-03	4.0728
		1e-04	4.4085
		1e-05	4.4368
	10	1e-03	7.9822
		1e-04	8.1265
		1e-05	8.2661
	100	1e-03	4.8385
		1e-04	6.0961
		1e-05	7.3100
Drug feature: ECFP<br>Cell feature: MUT_{sub}<br>Drug encoder: DNN<br>Cell encoder: DNN<br>Fusion module: DNN	1	1e-03	3.9342
		1e-04	4.0080
		1e-05	4.2700
	10	1e-03	7.8395
		1e-04	8.4977
		1e-05	8.7711
	100	1e-03	4.8417
		1e-04	7.5452
		1e-05	8.0381
Drug feature: ECFP<br>Cell feature: CNV_{all}<br>Drug encoder: DNN<br>Cell encoder: DNN<br>Fusion module: DNN	1	1e-03	3.9940
		1e-04	4.1404
		1e-05	4.3815
	10	1e-03	8.1826
		1e-04	8.9632
		1e-05	9.1461
	100	1e-03	4.7170
		1e-04	5.2934
		1e-05	6.6904
Drug feature: ECFP<br>Cell feature: CNV_{sub}<br>Drug encoder: DNN<br>Cell encoder: DNN<br>Fusion module: DNN	1	1e-03	4.1846
		1e-04	4.1801
		1e-05	4.3113
	10	1e-03	7.6436
		1e-04	8.1995
		1e-05	8.3756
	100	1e-03	5.1225
		1e-04	5.5915
		1e-05	5.7650

Continued Table S7: Leave-drug-out performance on GDSC2 with different learning rates

Framework	Random seed	Learning rate	MSE
Drug feature: ECFP<br>Cell feature: EXPall<br>Drug encoder: DNN<br>Cell encoder: DNN<br>Fusion module: DNN	1	1e-03	4.3534
		1e-04	4.8932
		1e-05	5.0718
	10	1e-03	7.8374
		1e-04	7.9301
		1e-05	8.0565
	100	1e-03	3.7370
		1e-04	8.8895
		1e-05	8.9421
Drug feature: ECFP<br>Cell feature: EXPsub<br>Drug encoder: DNN<br>Cell encoder: DNN<br>Fusion module: DNN	1	1e-03	3.5680
		1e-04	3.2701
		1e-05	3.2821
	10	1e-03	7.7290
		1e-04	7.8932
		1e-05	7.9804
	100	1e-03	4.4394
		1e-04	6.6563
		1e-05	6.8318
Drug feature: ECFP<br>Cell feature: EXPall<br>Drug encoder: DNN<br>Cell encoder: CNN<br>Fusion module: DNN	1	1e-03	4.6142
		1e-04	8.0166
		1e-05	8.8597
	10	1e-03	7.7014
		1e-04	7.9498
		1e-05	8.1231
	100	1e-03	4.0424
		1e-04	7.3359
		1e-05	7.7720
Drug feature: ECFP<br>Cell feature: EXPall<br>Drug encoder: DNN<br>Cell encoder: DAE<br>Fusion module: DNN	1	1e-03	4.3149
		1e-04	4.3784
		1e-05	4.4140
	10	1e-03	7.6099
		1e-04	8.4249
		1e-05	8.6134
	100	1e-03	4.7309
		1e-04	10.1454
		1e-05	10.3184

Continued Table S7: Leave-drug-out performance on GDSC2 with different learning rates

Framework	Random seed	Learning rate	MSE
Drug feature: SMILES<br>Cell feature: EXP_{all}<br>Drug encoder: CNN<br>Cell encoder: DNN<br>Fusion module: DNN	1	1e-03	3.7949
		1e-04	4.4906
		1e-05	4.8547
	10	1e-03	5.6447
		1e-04	6.1918
		1e-05	6.3139
	100	1e-03	5.1863
		1e-04	6.3772
		1e-05	6.6085
Drug feature: Graph<br>Cell feature: EXP_{all}<br>Drug encoder: A-FP<br>Cell encoder: DNN<br>Fusion module: DNN	1	1e-03	3.6163
		1e-04	3.9837
		1e-05	4.3046
	10	1e-03	7.3173
		1e-04	9.1388
		1e-05	9.8825
	100	1e-03	5.0116
		1e-04	6.6347
		1e-05	8.8488
Drug feature: Graph<br>Cell feature: EXP_{all}<br>Drug encoder: MPG<br>Cell encoder: DNN<br>Fusion module: DNN	1	1e-03	4.7418
		1e-04	5.8536
		1e-05	7.2577
	10	1e-03	5.0652
		1e-04	7.1137
		1e-05	7.6405
	100	1e-03	7.8333
		1e-04	8.8950
		1e-05	9.3134
Drug feature: Graph<br>Cell feature: EXP_{all}<br>Drug encoder: MPG<br>Cell encoder: DNN<br>Fusion module: MHA	1	1e-03	4.1910
		1e-04	5.0898
		1e-05	5.6642
	10	1e-03	8.8457
		1e-04	9.3129
		1e-05	9.6800
	100	1e-03	4.1297
		1e-04	4.5708
		1e-05	5.0960

Reference

[1] Li P, Wang J, Qiao Y, et al. An effective self-supervised framework for learning expressive molecular global representations to drug discovery[J]. Briefings in Bioinformatics, 2021, 22(6): bbab109.

[2] Shen B, Feng F, Li K, et al. A systematic assessment of deep learning methods for drug response prediction: from in vitro to clinical applications[J]. Briefings in Bioinformatics, 2023, 24(1): bbac605.

[3] Chawla S, Rockstroh A, Lehman M, et al. Gene expression based inference of cancer drug sensitivity[J]. Nature communications, 2022, 13(1): 5680.

[4] Jia P, Hu R, Pei G, et al. Deep generative neural network for accurate drug response imputation[J]. Nature communications, 2021, 12(1): 1740.
